# Dataset of factors impacting second language learning from Teachers' experience

**DOI:** 10.1016/j.dib.2021.107015

**Published:** 2021-03-27

**Authors:** Amaya Arigita-García, Roberto Sánchez-Cabrero, José Luis Estrada-Chichón

**Affiliations:** a Alfonso X El Sabio University, Spain; b Cádiz University, Spain

**Keywords:** Language, Second Language, Language Teaching, Second Language Learning

## Abstract

Developing an accurate second language competence different from the mother tongue has become an essential skill in today's globalized world, and therefore it is a highly valued and demanded learning among the main educational institutions and models. However, it is a complex process that is influenced by numerous and, in many cases, unknown factors, which are not usually taken into consideration when designing second language learning processes, which tend to lead to inadequate teaching and may lead to school failure that could have been avoided.

216 in-service teachers from all non-university educational stages of the Community of Madrid, Spain, evaluate the significance of 44 factors traditionally associated with second language learning, which are grouped into four general categories (factors linked to students; factors linked to teachers; learning structure and organisation; and learning environment) through a five-point Likert scale.

The data were collected using a *Google Forms* questionnaire through the research described in Arigita-García et al. (2021) [1]. The sample is heterogeneous concerning different attribute variables such as age, teaching experience, gender, school ownership, and the language in which classes are taught. The sample was obtained through social networks and teacher forums.

The data collection offers essential information to better understand the process of second language learning, as it gathers the experience and learning accumulated by the teachers who took part in this work, which implies direct information from the educational reality that they are intended to improve.

## Specifications Table

**Table:** 

Subject	Linguistics and Language
Specific subject area	Second Language Learning, Education
Type of data	Table Chart
How data were acquired	Computerized questionnaire through the Google Forms private server [2]
Data format	Raw
Parameters for data collection	The participants were in-service teachers of all non-university teaching stages in the Spanish non-university context who answered all the questions included in an online questionnaire.
Description of data collection	An individual and anonymous online questionnaire to 216 teachers describing the significance of 44 factors when learning a second language on a five-point Likert scale.
Data source location	Community of Madrid, Spain Latitude: N40°24′59.4′′ Longitude: W3°42′9.22′′
Data accessibility	https://doi.org/10.5061/dryad.zcrjdfnb4[3]
Related research article	A. Arigita-García, R. Sánchez-Cabrero, A. Barrientos-Fernández, L. Mañoso-Pacheco, F.J. Pericacho-Gómez, Pre-eminence of determining factors in second language learning: an educator's perspective from Spain, *Heliyon*. 7(2), e06282. [1]

## Value of the Data

• Second language teaching is a complex and little-known process. For this reason, the collection of first-hand experiences and knowledge acquired by 216 teachers from all education stages provides new and valuable information. This allows us to delve deeper into this field of study, which other studies in this area can benefit from.
• The data collection shows and compares the most significant factors involved in the process of second language learning, which is a piece of essential knowledge for the design of new and more efficient educational experiences in this field of study.
• The data collected make it possible to identify the needs, opportunities and shortcomings observed by teachers when teaching a second language. This information is extremely valuable to properly plan teachers’ future training and to test it in other studies.
• The information provided makes it possible to compare second language learning with research described in other studies, such as the learning of mathematical and cultural concepts, experimental sciences, natural sciences, etc.
• The data collected can be used by other studies to define the recommended profile for teachers at each educational stage and to determine the processes involved in teaching when developing students' educational curricula.

## 1 Data Description

**Fig. 1:**
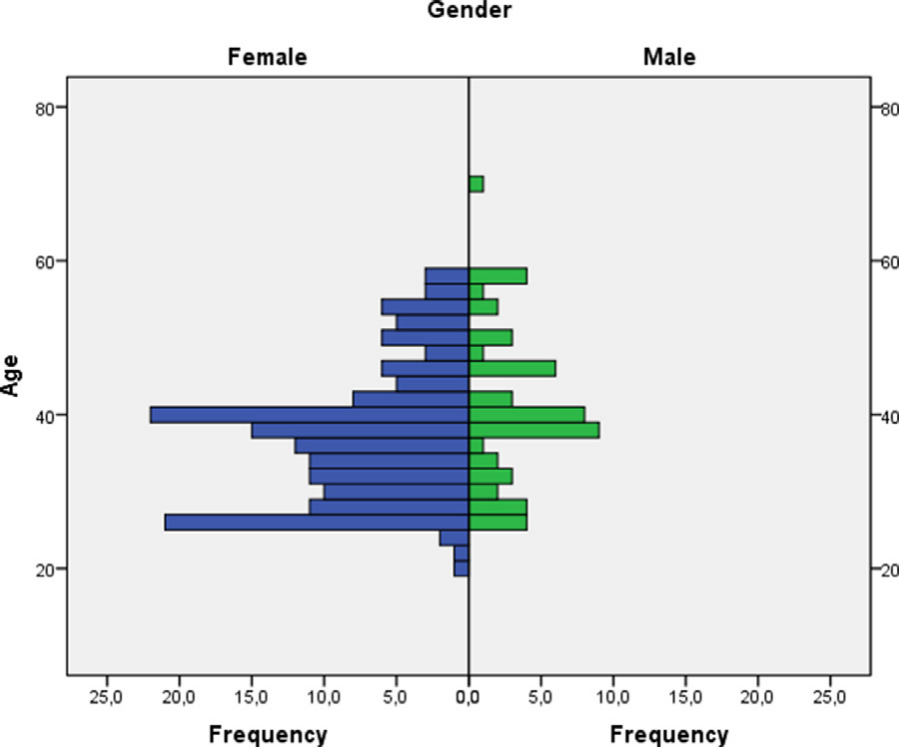
Age and gender pyramid.

**Fig. 2:**
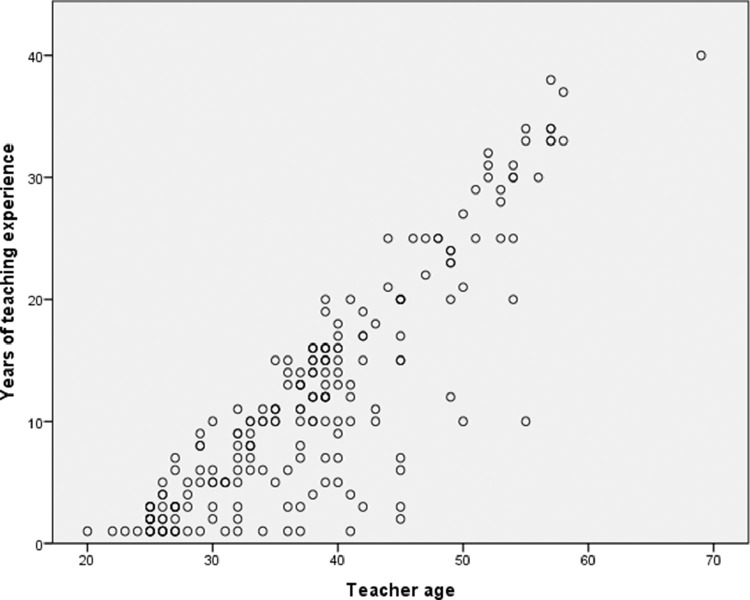
Years of teaching experience and teacher age scatter chart.
• Gender: nominal dichotomous qualitative variable (See Fig. 1).
• Years of teaching experience: discrete quantitative variable (See Fig. 2).
• Regional Education Authorities (DAT, Spanish acronym): nominal qualitative variable. It is distributed in five categories (East, West, North, South or capital) (See Fig. 3).

The data collected describe the valuation made by 216 teachers from the Community of Madrid, Spain, about the significance they perceive in 44 factors traditionally associated with second language learning [4], [5]. Raw data are presented here, without any manipulation on the part of the authors, using a *.xlsx file* (*Microsoft Excel format*) [3]. The 216 participants in the study constitute a representative sample of the population of non-university teachers in the Spanish educational context since they present great heterogeneity in different significant attribute variables [1]. The attribute variables considered in this study are:

• Teachers' age: discrete quantitative variable (See Figs. 1 and 2).

**Fig. 3:**
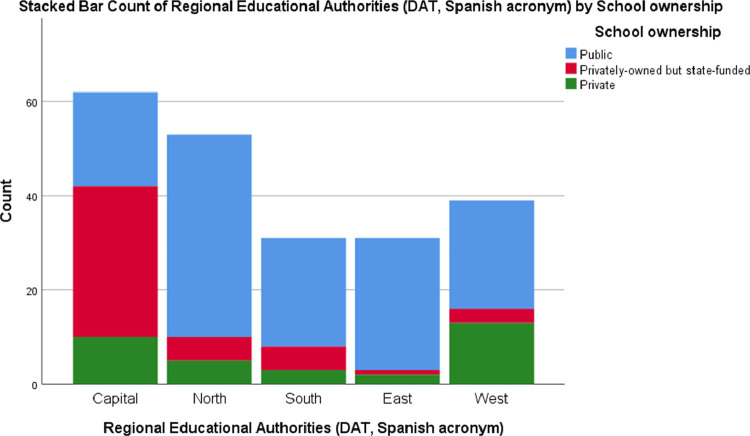
Stacked Bar Count of Regional Educational Authorities (DAT, Spanish acronym) by School ownership.
• School ownership: nominal qualitative variable. It is distributed in 3 categories (public, privately-owned but state-funded, and private) (See Fig. 4).

**Fig. 4:**
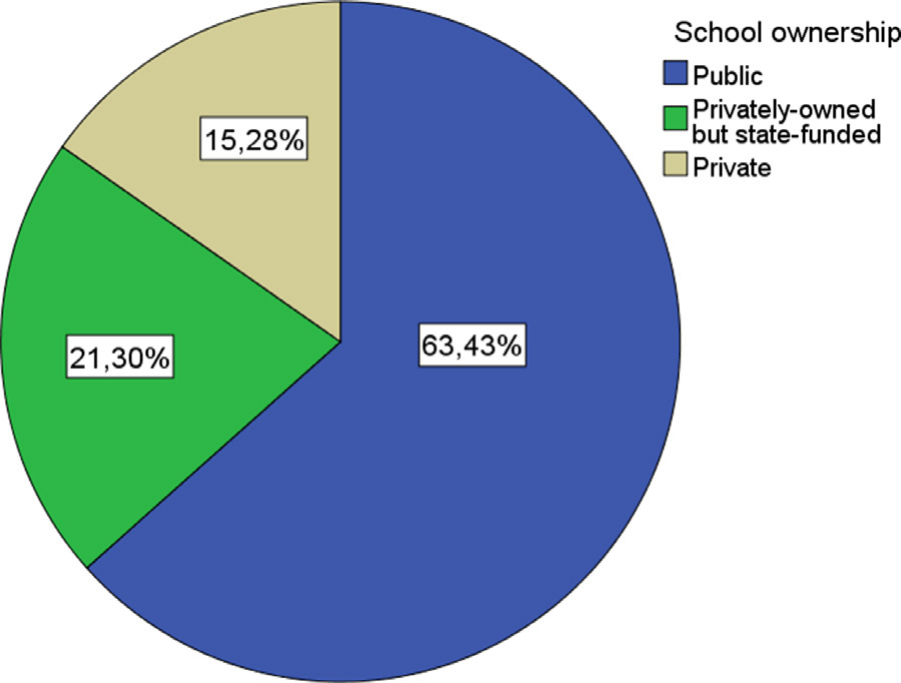
School ownership pie chart.
• Highest level of education taught: nominal qualitative variable with 3 categories (Infant Education (up to 6 years); Primary Education (from 6 to 12 years); and Secondary Education (from 12 to 18 years) (See Fig. 5).

**Fig. 5:**
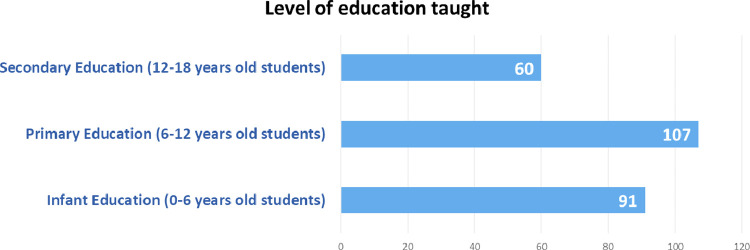
Level of education taught distribution.
• Teaching according to a bilingual educational programme: nominal dichotomous qualitative variable (See Fig. 6).

**Fig. 6:**
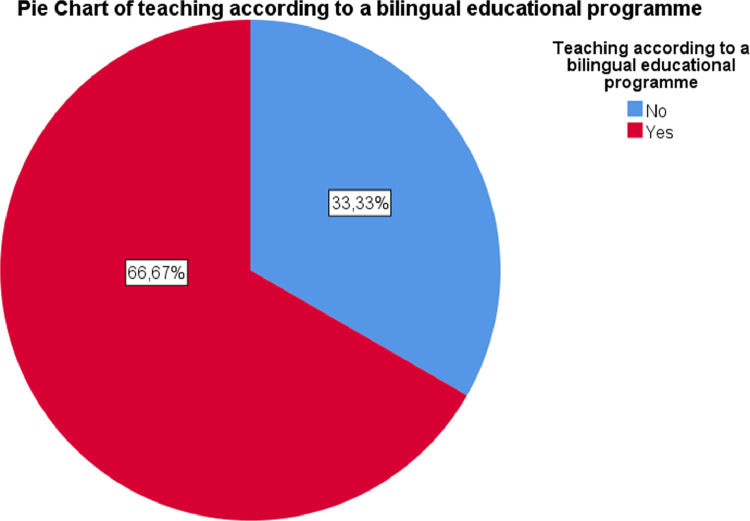
Pie Chart of teaching according to a bilingual educational programme.
• Language of instruction: nominal qualitative variable with 3 categories (teaching in the students’ mother tongue; teaching in the second language (English); or teaching in both languages) (See Fig. 7).

**Fig. 7:**
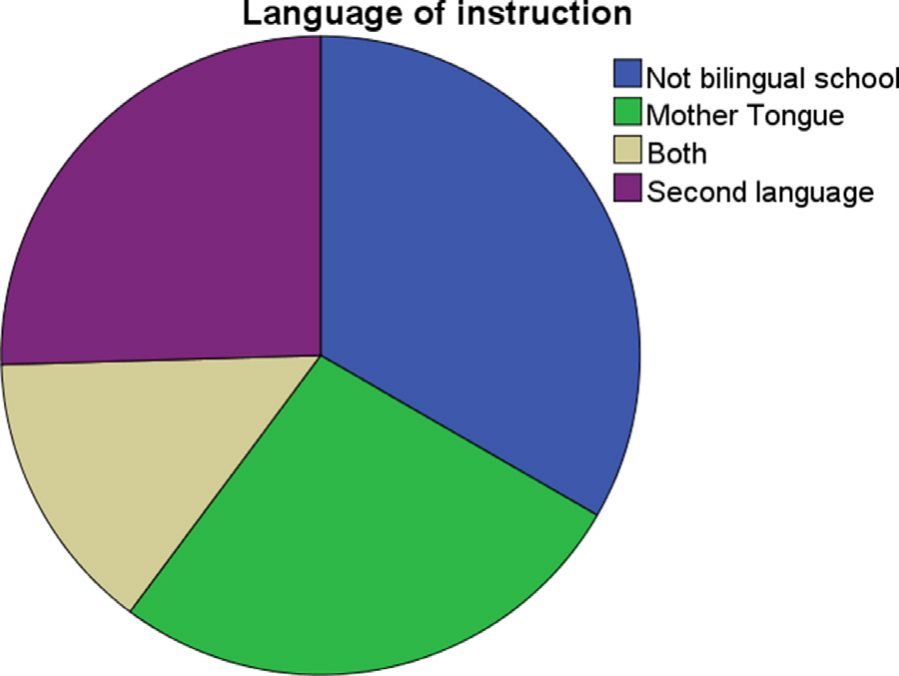
Language of instruction pie chart.

Each of the 44 factors linked to second language learning was measured through a five-point Likert scale, assigning to each statement a range of points ranging from -1- unlikely to -5- highly likely. Thus, it was possible to establish comparisons between each item in a simple way.

**Fig. 8:**
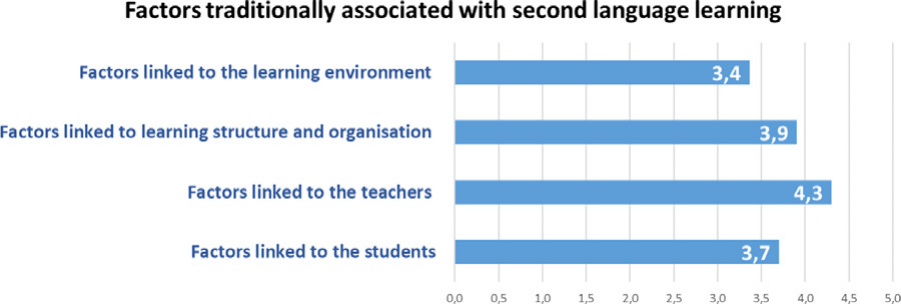
Factors traditionally associated with second language learning distribution.

The 44 factors are based on previous literature on second language teaching and learning [6], [7], [8], [9], compiled through the study by Arigita-García et.al. (2021) [1]. The 44 factors were grouped into 4 thematic categories (See Fig. 8), which are also based on previous relevant scientific work, are as follows: factors linked to the students [10,11]; factors linked to the teachers [12,13]; the learning structure and organisation [14]; and the learning environment [15,16].

The variables concerning the teachers’ perception of the students, the teachers themselves, the learning structure and organisation, and the learning environment are the following:

1. Factors linked to the students: A continuous quantitative variable derived by the arithmetic mean resulting from the participants’ responses to 16 questions that could be linked to second language learning, which have in common the direct relationship with the characteristics of the students. Each of these questions is described below:
  • Developed strategies for language learning: The teachers evaluate the significance of second language learning regarding the complexity and appropriateness of the method chosen by the students to learn new language skills.
  • The motivation for learning the target language: The teachers evaluate the significance of the students’ motivation when learning another language.
  • Fear of communicating in another language: The teachers evaluate the significance of the students' fear of communicating in the second language as a drawback to learning.
  • Degree of confidence in the achievement of the objectives: The teachers evaluate the significance of the students’ self-confidence when learning a second language as personal reach achievement.
  • Age: The teachers evaluate whether the age of the students impacts on second language learning.
  • Degree of empathy with the target language: The teachers evaluate whether the students’ sympathy with the second language impacts on learning.
  • Degree of language competence in the mother tongue: The teachers evaluate whether the students’ competence in the mother tongue impacts on second language learning.
  • Degree of self-esteem: It is evaluated whether the teachers perceive that the students’ self-esteem impacts on second language learning and competence.
  • Previous language learning experience: It is evaluated whether, according to the teachers, having had previous language learning experience impacts on second language learning.
  • Degree of cognitive maturity: It is evaluated whether the teachers perceive that the students’ degree of cognitive maturity impacts on second language learning.
  • Classroom behaviour: The teachers evaluate whether the students’ mother tongue competence impacts on second language learning.
  • Personality: The teachers evaluate the significance of students' personality features when learning a second language.
  • The socio-economic and cultural levels of the family: The teachers evaluate whether the socio-economic and cultural origin of the students impacts on second language learning.
  • Degree of empathy with the monolingual community: The teachers evaluate whether the students’ sympathy with the community that speaks the second language impacts on second language learning.
  • Order of birth: It is evaluated whether, according to the teachers, the fact that the student is a single child or a member of a large family, and the order of birth concerning his or her siblings, impact on second language learning.
  • Gender: The teachers evaluate whether there are differences when learning a second language depending on if the student is a boy or a girl.
2. Factors linked to the teachers: Continuous quantitative variable derived by the arithmetic mean resulting from the participants’ responses to 9 questions that could be linked to second language learning, which have in common the direct relationship with the characteristics of the teachers (See Fig. 2). Each of these questions is described below:
  • Teacher-student relationship: The teachers evaluate the significance of the quality and positive emotional involvement existing between students and teachers when learning a second language [17,18].
  • Use of appropriate teaching resources and materials: It is evaluated whether the teachers consider that the selection of teaching resources and materials has an impact on the students’ second language learning.
  • Communication strategies used: It is evaluated whether, according to the teachers, there are differences in the students’ strategies to learning a second language according to the strategy used when communicating with them.
  • The language input that the students receive: The significance that the teachers provide to the number of terms, concepts, and meanings that the students receive during the teaching-learning process.
  • Didactic planning: It is evaluated whether it is significant for the teachers to carry out adequate planning before teaching starts to improve the students' results concerning second language learning.
  • Level of language proficiency in the target language: It is evaluated whether establishing a higher level of language proficiency as a learning objective impacts on the students’ learning.
  • Education: It is evaluated whether the teachers consider that their level of academic formation impacts on the students’ second language learning.
  • Regular evaluation of the programmed objectives: It is evaluated whether, according to the teachers, establishing concrete methods of regular evaluation regarding the learning objectives has a direct impact on second language learning.
  • Use of information and communication technologies: The teachers evaluate the development of the students’ learning concerning the use of new technologies could have in the teaching-learning process [19].
3. Factors linked to learning structure and organisation: A continuous quantitative variable derived by the arithmetic mean resulting from the participants’ response to 11 questions that could be linked to second language learning, which have in common the direct relationship with the learning structure and organisation carried out in the learning environment (See Fig. 4). Each of these questions is described below:
  • The intensity of exposure to the target language: It is evaluated whether, according to the teachers, a more intense or invasive educational methodology could impact on a greater, faster, or deeper second language learning.
  • The methodological approach used when teaching: The teachers evaluate whether there are differences in second language learning according to the educational approach used.
  • The number of instruction hours per day: It is evaluated whether, according to the teachers, there is a relationship between the number of hours of second language exposure per week and knowledge.
  • The context in which learning takes place: It is evaluated whether the context in which the learning environment takes place impacts the quality and quantity of the students’ learning.
  • Degree of coordination between teachers: It is evaluated the impact that the existence of proper coordination between teachers could have to improve second language learning.
  • Organization of curricular contents: It is evaluated the impact on learning that a coherent and logical organization of contents could have.
  • Existence of pedagogical advice: The teachers evaluate the impact of the participation of adequate pedagogical advice on the students' learning process.
  • Organization of the educational space: It is evaluated the impact that the place where the teaching-learning process develops has on second language learning.
  • The geographical location of the educational institution: It is evaluated whether the ecological or urban context impacts on second language learning.
  • School ownership: The teachers evaluate whether the fact that the educational institution is private, privately-owned but state-funded or public is a significant factor in second language learning.
  • Size of the educational institution: The teachers evaluate whether the number of students in the educational institution could impact on second language learning.
4. Factors linked to the learning environment: A continuous quantitative variable derived by the arithmetic mean resulting from the participants’ response to 8 questions that could be linked to second language learning, which have in common the direct relationship with the context where the teaching-learning process takes place (See Fig. 5). Each of the questions is described below:
  • The attitude of the family towards the target language: It is evaluated, according to the teachers, the impact of the families’ attitude towards the students’ second language learning
  • The attitude of the society towards the target language: It is evaluated whether the fact that society values positively the knowledge and skills in the second language can impact on learning.
  • The attitude of the educational institutions towards the target language: It is evaluated whether the respect that teachers perceive for educational institutions has a direct impact on second language learning.
  • Phylogenetic proximity between the mother tongue and the target language: It is evaluated whether the existence of phylogenetic roots between the mother tongue and the second language facilitates and improves the students’ learning process.
  • Coexistence of similar cultural elements: The impact on the learners that the mother tongue and the second language could share cultural meanings.
  • Presence of cultural stereotypes: It is evaluated whether the existence of cultural stereotypes both positive and negative linked to the second language could impact the students’ learning process.
  • The historical relationship between the mother tongue and the target language: The teachers evaluate whether the historical interaction between the two language communities can impact the students’ learning process.
  • The social distance between countries: It is evaluated whether the social proximity between the two language communities could have an impact on second language learning.

The data collected show excellent internal reliability (*α* = .886) after determining the *Cronbach Alpha coefficient*.

Finally, the normality of the sampling distribution according to the evaluated variables was checked to establish the selection or not of parametric analysis tests [1]. Only the distribution of the variable factors linked to the teachers has a different distribution than normal distribution about the *K-S test (0,096)*. Considering that the remaining variables show normal distributions and that the number of participants (*N=*216) is worth considering, it is assumed the normality of the results of the study and, therefore, it is possible to analyze the results obtained through parametric statistical tests. However, the decision depends on the researchers who use this data.

## 2 Experimental Design, Materials and Methods

In order to design the research tool (online questionnaire designed *ad hoc)*, a review of available literature was conducted [6], [7], [8], [9]; those factors believed to be influential in second language learning or foreign language learning by previous researches on this area were collected and combined [1]. It was assumed that the characteristics of the student body may vary depending on the Regional Educational Authority (DAT) evaluated, ownership model chosen by parents or tutors and schooling stage. To ensure that the questionnaire was analysed from the broadest possible perspective, it was enlisted the help of a group of 45 teachers (29 women and 16 men). Each teacher represented one of the five DAT in the Community of Madrid (East, West, North, South or Capital), one of the three ownership models currently in place in the same area (state-owned, private or privately-owned but state-funded), and one of the three stages of mandatory schooling (Infant, Primary or Secondary). Finally, to ensure that the knowledge of the topic being analysed was as uniform as possible, we decided that the teachers enlisted would have a teaching experience equal to or over five years of professional development.

The questionnaire could only be initiated once the participants were told about the objectives of the research, their rights, and once they confirmed their participation by providing informed consents to be part of the research, as set out in the *Declaration of Helsinki* in 2013 [20]. The first eight questions included the attribute variables of the participants. The questionnaire contained 44 questions to be answered on a five-point Likert scale [2].

The questionnaire was completed by 216 teachers through the *Google Forms tool* on an individual basis [2]. The data collection process was carried out during the academic year 2016-2017. It was disseminated online among the teachers of Infant, Primary and Secondary Education of public, privately-owned but state-funded and private educational institutions of the Community of Madrid, Spain. The sample was obtained through teachers' online forums and the `snowball' sampling technique. In November 2017, the data collection period was closed, and the data were transformed into *.xlsx file* (*Microsoft Excel format*) [3].

## Ethics Statement

The participation in this research could only be initiated once the participants were told about the objectives of the research, their privacy rights and once they confirmed their participation by providing informed consents to be part of the research, as set out in the *Declaration of Helsinki* in 2013 [20].

## CRediT Author Statement

**Amaya Arigita-García:** Conceptualization, Investigation, Research tool, Supervision, Writing- Reviewing and Editing; **Roberto Sánchez-Cabrero:** Visualization, Methodology, Data curation, Writing - Original draft preparation, Writing - Reviewing and Editing; **José Luis Estrada-Chichón:** Investigation, Supervision, Validation, Writing - Reviewing and Editing, Translation.

## Declaration of Competing Interest

The authors declare that they have no known competing for financial interests or personal relationships which have, or could be perceived to have, influenced the work reported in this article.
